# Producing Lignin-Based Polyols through Microwave-Assisted Liquefaction for Rigid Polyurethane Foam Production

**DOI:** 10.3390/ma8020586

**Published:** 2015-02-10

**Authors:** Bai-Liang Xue, Jia-Long Wen, Run-Cang Sun

**Affiliations:** 1 Beijing Key Laboratory of Lignocellulosic Chemistry, Beijing Forestry University, Beijing 100083, China; E-Mails: xuebailiang@bjfu.edu.cn (B.-L.X.); wenjialong@bjfu.edu.cn (J.-L.W.); 2 State Key Laboratory of Pulp and Paper Engineering, South China University of Technology, Guangzhou 510641, China

**Keywords:** microwave heating, lignin, liquefaction, Polyurethane (PU) foam

## Abstract

Lignin-based polyols were synthesized through microwave-assisted liquefaction under different microwave heating times (5–30 min). The liquefaction reactions were carried out using polyethylene glycol (PEG-400)/glycerol as liquefying solvents and 97 wt% sulfur acid as a catalyst at 140 °C. The polyols obtained were analyzed for their yield, composition and structural characteristics using gel permeation chromatography (GPC), Fourier transform infrared (FT-IR) and nuclear magnetic resonance (NMR) spectra. FT-IR and NMR spectra showed that the liquefying solvents reacted with the phenol hydroxyl groups of the lignin in the liquefied product. With increasing microwave heating time, the viscosity of polyols was slightly increased and their corresponding molecular weight (*M_W_*) was gradually reduced. The optimal condition at the microwave heating time (5 min) ensured a high liquefaction yield (97.47%) and polyol with a suitable hydroxyl number (8.628 mmol/g). Polyurethane (PU) foams were prepared by polyols and methylene diphenylene diisocyanate (MDI) using the one-shot method. With the isocyanate/hydroxyl group ([NCO]/[OH]) ratio increasing from 0.6 to 1.0, their mechanical properties were gradually increased. This study provided some insight into the microwave-assisted liquefied lignin polyols for the production of rigid PU foam.

## 1. Introduction

Lignin, as a nontoxic, low-cost and renewable resource, has been considered as a substitute for some petrochemical products to mitigate the effects of the petroleum resource crisis and environmental pollution caused by non-biodegradable polymers. Currently, an enormous amount of lignin is produced as a co-product in the pulping and papermaking industry. However, it is primarily used as fuel, and only a small amount of lignin (1%–2%) is separated and utilized for value-added products, including stabilizers, dispersants and surfactants [1,2,3].

Polyurethane (PU), one of the most widely-used synthetic polymers for its versatility, is usually prepared from the formation of urethane linkages by the reaction of isocyanate with a polyether or polyester polyol. However, one of the problems related to the production of PU is its dependence on petroleum-based resources. Lignin contains high concentrations of hydroxyl groups on the aromatic macromolecule. Therefore, it can function as the polyols to form the PU structure [4,5]. In order to improve the performance of lignin in PU formulation, extensive attention has been paid to the study of the reactivity of specific functional groups in lignin as polyol precursors through lignin modification, instead of using underivatized lignin directly [6,7,8,9].

Nowadays, replacing of the polyols derived from petroleum-based materials with the polyols from renewable resources, such as lignin, is having a deep impact on the PU foam industry. One of the techniques to obtain the polyols from renewable resources is liquefaction. Various lignocellulosic materials, such as wood [10], paper [11], straw [12], bamboo [13], sugar cane [14] and corn stover [15], have been liquefied in different solvents to generate polyols and to subsequently produce the PU foam. However, there is very limited information about the liquefaction of lignin in the literature, to our best knowledge. Jin* et al.* [16] reported that the enzymatic hydrolysis of lignin can be liquefied with polyethylene glycol (PEG) and glycerol, and the influences of the liquefaction parameters on the residue content and hydroxyl number of the liquefaction product were fully discussed, suggesting that the hydroxyl number of the liquefaction product was increased compared with those of the polyols.

Microwave heating is an alternative method to direct the microwave energy into the target object, due to the applied electromagnetic field. Compared with conventional heating, microwave heating penetrates and simultaneously heats the bulk of the material and, therefore, reduces the reaction time [17]. Most research about biomass liquefaction in the past was performed under conventional heating conditions, such as oil bath, salt bath and electrical furnace. The application of microwave irradiation to liquefy wood into polyols was not reported until very recently [18,19,20,21,22,23,24]. The liquefaction of lignin with polyols under microwave heating was also investigated [25]. Therefore, this study profoundly looks into the liquefaction behavior of lignin into polyols under microwave heating for the preparation of rigid PU foam.

Based on previous studies [16,25], the optimum conditions were selected in microwave heating liquefaction using the mixed liquefying reactants of PEG-400 and glycerol (80/20), lignin/liquefying reactants at a ratio of 0.2, reaction temperature at 140 °C and catalyzed with 1.5% sulfuric acid (based on the weight of PEG-400 and glycerol). In order to obtain the polyols under the optimal microwave heating condition, the effects of the microwave heating time on the properties of the polyols were fully investigated through Fourier transform infrared (FT-IR), gel permeation chromatography (GPC), ^1^H, ^13^C and ^31^P nuclear magnetic resonance (NMR) spectra. Moreover, the optimal polyols obtained were used to evaluate the performances of the PU foam at different [NCO]/[OH] ratios.

## 2. Results and Discussion

### 2.1. Liquefaction Yield and Viscosity of the Liquefied Products

The liquefaction yield as a function of microwave heating time is summarized in Table 1. The liquefaction yield increased with increasing microwave heating time from 5 to 20 min; however, the liquefaction yield was slightly reduced when the microwave heating time was 30 min. This could be attributed to the recondensation polymerization of the lignin fragments during the liquefaction process [26]. In contrast with the recently published literature, Jin* et al.* [16] reported that the optimal liquefaction yield under conventional heating condition was 98.4%; unfortunately, the hydroxyl values of the liquefied products were relatively low for preparing the rigid PU foam, and the liquefaction time lasted as long as 60 min. Sequeiros* et al.* showed that the optimal liquefaction yield under microwave heating could reach up to 99.07% [25]; however, the reaction temperature was 155 °C. Accordingly, 20 min was the optimal reaction time for microwave-assisted liquefaction of the lignin, with a maximum liquefaction yield of 98.75%. However, the fact should be considered that shorter times in the microwave-assisted liquefaction than conventional reactions contribute to the process flexibility for the development of a continuous liquefaction reactor [27]. Furthermore, the microwave heating time did not cause a significant influence on the liquefaction yield; thus, 5 min could be selected as the alternative microwave heating time.

**Table 1:** Liquefaction conditions, yield and viscosities of the polyols. P, polyol.

Sample	Microwave Heating Time (min)	Liquefaction Yield (%)	Viscosity (mPa·s)
P5	5	97.47%	1035
P10	10	98.34%	1116
P20	20	98.75%	1161
P30	30	97.19%	1266

The viscosity of the polyols is another critical factor for the preparation of rigid PU foam. High viscosity could cause problems when mixing the foam ingredients and affect the generation and distribution of the cells formed by the CO_2_ during the polymerization reaction [28]. The effect of microwave heating time on the viscosity is shown in Table 1. With the microwave heating time increasing from 5 to 30 min, the corresponding viscosity of the polyols increased slowly from 1035 to 1266 mPa·s. This could be attributed to the greater degradation of lignin in the polyol with increasing microwave heating time. Therefore, the microwave heating time (5 min) could be used as the alternative condition to liquefy the lignin.

### 2.2. Molecular Weight of the Liquefied Products

Weight-average (*Mw*) and number-average (*Mn*) molecular weights and polydispersity (*Mw*/*Mn*) of the liquefied products are shown in Table 2. As compared with the lignin, the molecular weight of all the liquefied products obtained by microwave-assisted liquefaction drastically declined, which was due to the fact that the microwave heating decomposed lignin components into substances with relatively lower molecular weight. The decrease of the molecular weight was also due to the incorporation of a large amount of PEG and glycerol. When the microwave heating is applied in the liquefaction reaction, it is interesting to note that the molecular weight of the liquefied products increased with increasing microwave heating time. These results probably originated from two aspects: on the one hand, a condensed structure was formed by the incorporation of the aliphatic glycerol and PEG moieties into the lignin structure [29]; on the other hand, a self-polymerization reaction occurred during the liquefaction reaction of the dissolved lignin by glycerol and PEG. In short, in the microwave-assisted liquefaction process, the lignin was degraded to small fragments of lower molecular weight in the presence of sulfuric acid under high temperatures. Then, the reaction of the hydroxyl groups in the fragments with PEG formed the ether bonds in the liquefied products. Finally, the fragments recondensed and formed the residue. Therefore, the microwave heating time (5 min) could be used as the alternative liquefaction condition of the lignin for producing more hydroxyl groups.

**Table 2:** Weight-average (*Mw*) and number-average (*Mn*) molecular weights and polydispersity (*Mw*/*Mn*) of the four polyols.

Entry	Lignin	Polyol Type ^a^			
		P5	P10	P20	P30
*Mw*	2792	525	673	725	1108
*Mn*	909	461	467	480	456
*Mw/Mn*	3.07	1.13	1.44	1.51	2.43

^a^ Corresponding to the polyol type in Table 1.

### 2.3. FT-IR Analysis for the Liquefied Products

**Figure 1:**
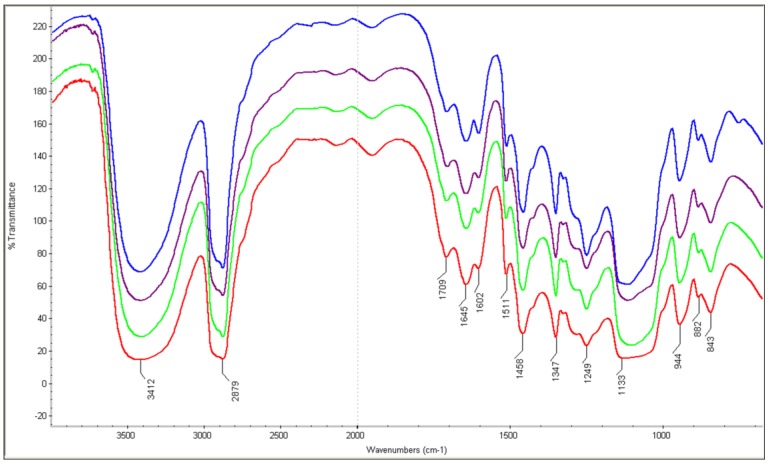
FT-IR spectra of all of the liquefied products.

The FT-IR spectra of the liquefied products are shown in Figure 1. The bands around 3412 cm^−1^ (O–H stretching), 2879 cm^−1^ (C–H stretching), 1036 cm^−1^ (C–O stretching) and 1709 cm^−1^ (C=O stretching vibration) exist in the liquefied products [30]. It was clear that the chemical structure of the liquefied products did not change with increasing microwave heating time.

The relative intensities of the bands at 1602 and 1511 cm^−1^ attributed to the skeleton vibration of the benzene ring in lignin did not change in all of the liquefied products. According to the conclusion of Jin* et al.* [16], the potential reason was that the breakdown of the bonds only appeared between the couplings of the benzene ring, and a smaller benzene ring unit was probably formed. These results are in accordance with the corresponding molecular weight results. The strong absorption peak at 1458 cm^−1^ belongs to the symmetric distortion vibration of methylene, which might be due to the structure of PEG and glycerol. It should be pointed out that the absorption peak of C–O in the phenol hydroxyl groups of the lignin shifted from 1220 to 1249 cm^−1^, which indicated that the phenol hydroxyl groups of the phenylpropane structure in the lignin reacted with the liquefied solvents in the final product [16,25]. Furthermore, the effects of the microwave heating time on the hydroxyl group and the acid value of the liquefied products were further investigated by various NMR techniques.

### 2.4. ^1^H and ^13^C NMR Analysis for the Liquefied Products

**Figure 2:**
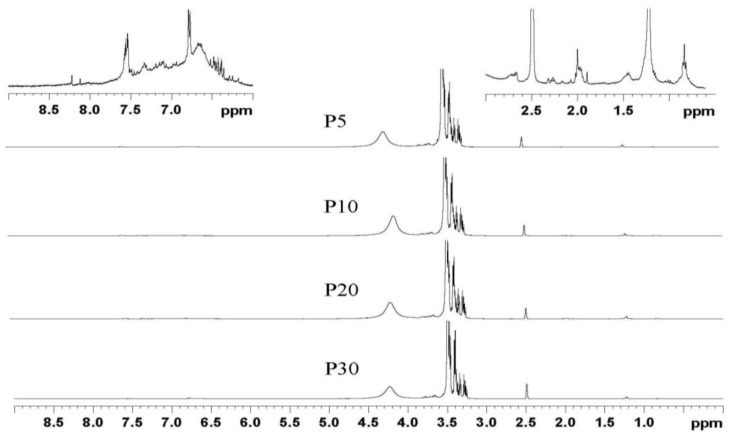
^1^H NMR spectra of all the liquefied products.

The ^1^H NMR spectra of the liquefied products are shown in Figure 2. It was clear that the microwave heating time did not have significant effects on the characteristic peaks of the liquefied lignin polyols. The small peaks in the aromatic region (6.5−8.0 ppm) indicated that the essential feature of the lignin did not change in the liquefaction process. In the 0.5−2.5 ppm region, these assignments are attributed to the methyl and methylene from the structure of PEG and glycerol. The strong peaks in the region (3.0−4.0 ppm) are mainly due to the PEG or glycerol molecules. These results are further corroborated by the ^13^C NMR spectra.

**Figure 3:**
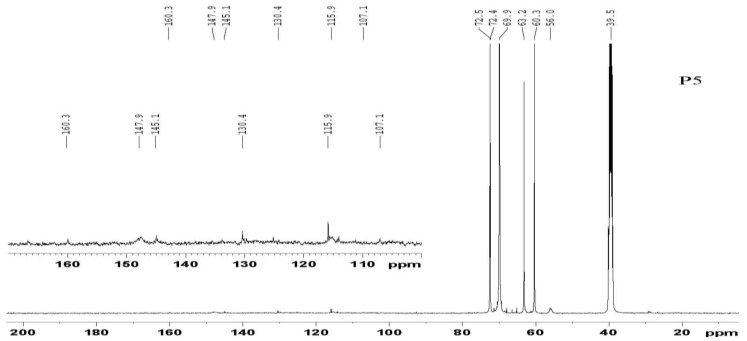
^13^C NMR spectrum of Sample P5.

**Figure 4:**
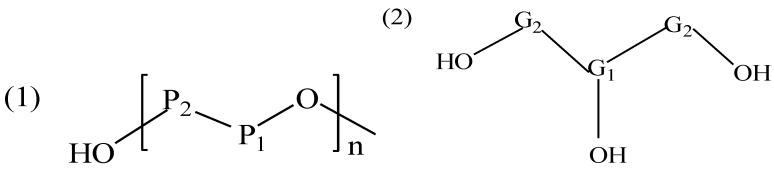
Structure nomenclature for NMR assignments of (1) PEG-400 and (2) glycerol.

A representative ^13^C NMR spectrum of Sample P5 (P, polyol) is shown in Figure 3. The assignments of secondary carbons (P_1_) and primary carbons (P_2_) (Figure 4) within PEG are 69.9 and 60.3 ppm, respectively. The intense peaks at 72.5 and 63.2 ppm are attributed to the primary carbons (G_1_) and secondary carbons (G_2_) of glycerol, respectively [31]. In the 140–150 ppm region, small peaks are consistent with aromatic ether carbons, such as the condensation product between the phenolic hydroxyl groups of lignin and PEG/glycerol. All these results are well in accordance with the analysis of FT-IR.

### 2.5. ^31^P NMR Analysis for the Liquefied Products

The ^31^P NMR method, based on a hydroxyl group reacting with a phosphitylation reagent, is used to quantify several different types of the hydroxyl groups in the liquefied products. The phosphitylation agent, 2-chloro-4,4,5,5-tetramethyl-1,3,2-dioxaphospholane (TMDP), has been used widely for quantitatively analyzing lignin [32], carbohydrates [33], as well as liquefied (bark) biomass polyols [31], but has not been applied previously to characterize the liquefied lignin products.

**Figure 5:**
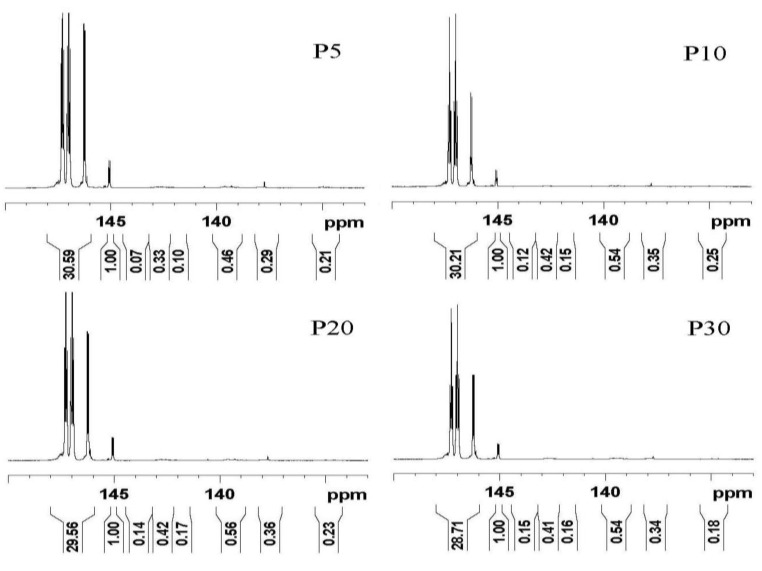
^31^P NMR spectra of all the liquefied products.

The ^31^P NMR spectra of all of the liquefied products are shown in Figure 5, and the corresponding hydroxyl values are summarized in Table 3. The peaks of the hydroxyl groups at 147.3 and 146.2 ppm are attributed to G_2_ and G_1_ in glycerol, respectively. The peak at 147.0 ppm is assigned to P_2_ in the hydroxyl groups of PEG [34]. The content of hydroxyl groups was obtained by integration of the following spectral regions: aliphatic hydroxyls (149.1–145.0 ppm), condensed syringyl phenolic units (144.5–143.3 ppm), non-condensed syringyl phenolic units (143.3–142.0 ppm), condensed guaiacyl phenolic hydroxyls (142.0–141.5 ppm), non-condensed guaiacyl phenolic hydroxyls (140.5–138.6 ppm), *p*-hydroxyphenyl phenolic units (138.5–137.3 ppm) and carboxylic acids (135.9–134.0 ppm) [35,36]. It was clear that all the samples (P5–P30) were similar in their total hydroxyl content and simultaneously had a typical hydroxyl value used in making the rigid PU foam. One expected result was that the shortest microwave heating time (5 min) could provide the optimal hydroxyl value for the microwave-assisted lignin liquefaction.

**Table 3:** Quantification of the lignin-derived polyols by the quantitative ^31^P-NMR method.

Polyol Type ^a^	Aliphatic OH	Syringyl OH		Guaiacyl OH		*p*-Hydroxy Phenyl OH	Total OH	Carboxylic Group
		C^b^	N-C^c^	C	N-C^c^			
P5	8.289	0.019	0.089	0.027	0.125	0.079	8.628	0.057
P10	8.187	0.033	0.114	0.041	0.146	0.095	8.616	0.068
P20	8.011	0.038	0.114	0.046	0.152	0.098	8.459	0.062
P30	7.780	0.041	0.111	0.043	0.146	0.092	8.213	0.049

^a^ Corresponding to the polyol type in Table 1; C^b^, condensed; N-C^c^, non-condensed.

However, unexpectedly, aliphatic hydroxyl groups accounted for most of the hydroxyl groups. The loss of the phenolic hydroxyl groups may be due to their condensation reaction between the phenolic hydroxyl of lignin and PEG/glycerol, analogous to a chain extension. Furthermore, the liquefaction process of the lignin could convert the sterically-hindered phenolic hydroxyls into an accessible aliphatic hydroxyl, which could react readily with methylene diphenylene diisocyanate (MDI) in producing the rigid PU foam [31]. Therefore, microwave-assisted liquefaction of the lignin could be used as an alternative method to produce industrially-relevant polyols. In the present study, the optimal condition for the microwave heating liquefaction of lignin was carried out at 140 °C for 5 min.

### 2.6. Compressive Property of the Rigid PU Foam

**Figure 6:**
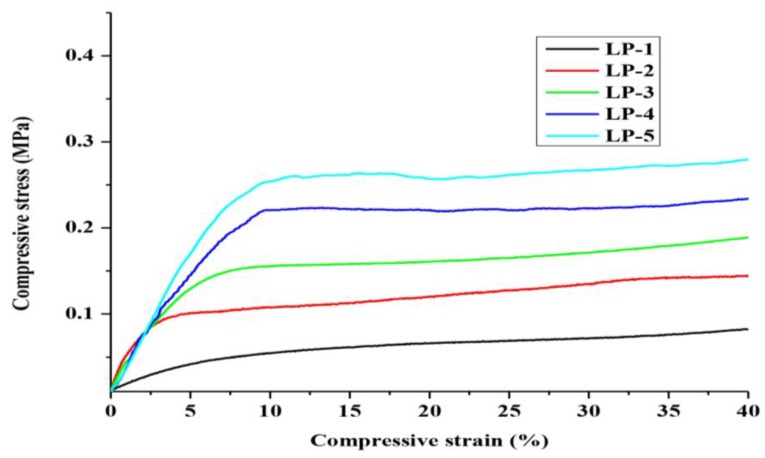
Mechanical properties of the rigid PU foams.

One of the most important parameters in the rigid PU foam preparation is the molar ratio of isocyanate to hydroxyl groups ([NCO]/[OH]). With the limited compression of all the samples (compressive strain of 40%), the mechanical properties of the resulting rigid PU foams in terms of compressive strength and strain, as influenced by the [NCO]/[OH] ratios, are shown in Figure 6.

With an increase of the [NCO]/[OH] ratio from 0.6 to 1.0, the initial slopes of all the LP samples firstly increased and reached their maximum (LP-5), then finally remained relatively constant. It can be seen that the flexibility of the PU foams had gradually decreased, while their rigidity gradually increased. This was due to the fact that the hard segment formed through the reaction between isocyanates and lignin-based polyols, as well as the crosslink density increased with an increase of the [NCO]/[OH] ratio [37]. The hard segment affects the mechanical characteristics of rigid PU foam, and a higher hard segment could increase the hardness of rigid PU foam, but simultaneously decrease its flexibility. Additionally, when the [NCO]/[OH] ratio of LP samples exceeds 0.6, their compressive strength is higher than 0.1 MPa, which is a sufficient value for many rigid PU foam applications [38]. Moreover, as compared to those synthesized from commercial sucrose and glycerol polyols, Samples LP-4 and LP-5 ([NCO]/[OH] ratio > 0.7) showed enhanced mechanical properties [39,40]. Thus, the microwave-assisted liquefaction is a promising heating method to produce the lignin-derived polyols for producing rigid PU foam products.

## 3. Experimental Section

### 3.1. Materials

Methyl diphenyl diisocyanate (MDI-50) with 50% 4,4- and 50% 2,4-isomers was supplied by Yantai Wanhua Co., Shandong, China. [NCO] (7.50 mmol/g) is the concentration of isocyanate groups in the MDI. Polyethylene glycol with a molecular weight of 400 g/mol (PEG-400) was used as the polyether polyol. Di-n-Butyltin dilaurate (DBTDL), a catalyst used in manufacturing polyurethane foam, was obtained from Sinopharm Chemical Reagent Co., Shanghai, China. AK-8801, a silicone surfactant used to stabilize the foam, was a commercial product from Dymatic Shichuang Chemical Co., Nanjing, China. Distilled water was used as a blowing agent. Lignin was extracted with mild alkaline solution from corncob residue after hydrolysis of hemicelluloses and was supplied by the Longlive Biological Technology Co., Shandong, China. The alkaline lignin contained 94.65% Klason lignin, 5.02% acid-soluble lignin and 0.33% polysaccharides [41]. All of these commercial products were used as received without any further pretreatment.

### 3.2. Microwave Heating Liquefaction Procedure

According to the published literatures [16,25], PEG-400 and glycerol were used as the liquefaction reagents at a ratio of 80:20 (*w*/*w*), and the weight ratio of liquefied solvents to lignin was 5. All of the reactions were catalyzed with 1.5% sulfuric acid, calculated based on the weight of liquefaction reagents (PEG-400 and glycerol). Microwave heating liquefactions were carried out in a microwave system (Ethos EX, Milestone, 25 Controls Drive, Shelton, CT, USA) equipped with an internal temperature sensor inserted directly into the glass flask. The typical liquefaction procedure for the experiments is shown as follows: 10 g lignin, 50 g PEG/glycerol and 0.75 g sulfuric acid catalyst were loaded into the glass flask and pre-mixed thoroughly by stirring before liquefaction. The liquefaction temperature was increased from room temperature to 140 °C at a heating rate of 20 °C/min and then was kept at 140 °C. After liquefaction for a preset time (5–30 min), the glass flask was immersed in cold water for ~10 min. Sodium hydroxide aqueous solution (40%) was added to neutralize the acid after liquefaction, and the neutralized liquefied lignin was stored in a refrigerator. The microwave-assisted liquefaction experiments were carried out for four different times (5, 10, 20 and 30 min). The corresponding lignin-based polyols were named P5, P10, P20 and P30, respectively.

### 3.3. Characterization of the Liquefied Polyols

#### 3.3.1. Determination of Liquefaction Yield

Liquefied product (1 g) was diluted with 20 mL 1,4-dioxane and water (4/1, *v*/*v*), and the dilution was adequately stirred over 4 h and vacuum-filtered through a filter disk. The solid residues were dried in an oven at 105 °C. The liquefaction yield was calculated as follows:

Yield = [1 – *M/M*_0_] × 100% (1)

where *M*_0_ is the mass of lignin and *M* is the mass of the solid residues after the liquefied product was dissolved in 1,4-dioxane and water.

#### 3.3.2. Viscosity and GPC Measurement

The viscosity of the liquefied products was determined using a Brookfield dial reading rotary viscometer (Model LVT). The reported data are the average of five measurements. The molecular weights of lignin and polyols were measured using a gel permeation chromatography system (GPC, Agilent 1200, Santa Clara, CA, USA) equipped with a PL-gel mixed bed high performance liquid Chromatography (HPLC) column (inner diameter: 7.5 mm; length: 300 mm; particle size: 10 um; mid-weight range: 500–10 M). Detection was achieved with a Knauer differential refractometer. The column was eluted with tetrahydrofuran at a flow rate of 1.0 mL/min. Samples (4 mg) were dissolved in 2 mL tetrahydrofuran. Monodisperse polystyrene was used as the standard for calibration.

#### 3.3.3. FT-IR Analysis

FT-IR spectra were recorded using a Thermo Scientific Nicolet iN10 FT-IR Microscope (Thermo Nicolet Corporation, Madison, WI, USA) equipped with a liquid nitrogen cooled MCT detector. Samples were ground and pelletized using BaF_2_, and their spectra were recorded in the range from 4000 to 700 cm^−1^ at 4-cm^−1^ resolution and 128 scans per sample.

#### 3.3.4. NMR Spectroscopy

The ^1^H and ^13^C NMR spectra of all of the liquefied products were acquired in dimethyl sulfoxide-d_6_ (DMSO-d_6_) at a concentration of 50 mg/mL. ^1^H NMR spectra were recorded using a 1-s relaxation delay, a pulse angle of 30°, an acquisition time of 3.98 s, a spectral width of 9600 Hz and 128 scans. The ^13^C NMR measurements were conducted using a 0.1-s relaxation delay, a pulse angle of 90°, an acquisition time of 1.42 s, spectral width of 35 kHz and 20,000 scans. Both were calibrated using tetramethylsilane (TMS) [9].

The quantitative ^31^P NMR spectra of all of the liquefied products were obtained using published procedures [33,35]. Samples (40 mg) were dissolved in 500 μL of anhydrous pyridine and deuterated chloroform (1.6:1, *v*/*v*) under stirring. This was followed by the addition of 100 μL of cyclohexanol (22.01 mg/mL), as an internal standard, and 50 μL of chromium (III) acetylacetonate solution (5.6 mg/mL in anhydrous pyridine and deuterated chloroform 1.6:1, *v*/*v*), as a relaxation reagent. Finally, the mixtures were treated with 100 μL of phosphitylation agent (TMDP) (2-chloro-4,4,5,5-tetramethyl-1,3,2-dioxaphospholane) and transferred into a 5 mm NMR tube for subsequent NMR analysis.

All NMR experiments were carried out on a Bruker AV III NMR spectrometer at 400 MHz at 25 °C. Spectra were processed and analyzed using the Bruker Topspin 2.1 software package (Bruker, Karlsruhe, Germany).

### 3.4. Preparation of the Rigid PU Foam

The PU foams were prepared by the one-shot method [41]. The mixtures of 15 g liquefied products, 0.3 g AK-8801, 0.3 g Di-n-butyltin dilaurate (DBTDL) and 0.5 g water were uniformly stirred in a paper cup at room temperature for 5 min. Afterwards, pre-determined MDI (14.38–23.96 g MDI; [NCO]/[OH] ratio, 0.6–1.0) was added into the paper cup and stirred vigorously with a high-speed mixer (2000 rpm) for 1 min. Finally, the polymerized mixture was quickly poured into a cubic paper container at room temperature to produce free-rise foam. The foams obtained were cured for 7 days at room temperature and then were conditioned for 24 h at 25 °C, 50% relative humidity before testing. The samples according to the [NCO]/[OH] ratio from 0.6 to 1.0 at 0.1 intervals were referred to as LP-1 to LP-5, respectively. The [NCO]/[OH] ratio is given as follows:

[NCO]/[OH] ratio = *M*_MDI_ × *W*_MDI_/(*M*_polyol_ × *W*_polyol_ + *W*_Water_ × 2/18 × 1000) (2)

where *M*_MDI_ (7.5 mmol/g) is the content of the isocyanate group in MDI, *M*_polyol_ is the content of the hydroxyl groups in the liquefied products and *W_MDI_*, *W*_polyol_ and *W*_water_ are the weights (g) of MDI, liquefied products and water, respectively [15].

### 3.5. Mechanical Properties of the Rigid PU Foams

The compressive properties of the rigid PU foams were measured at ambient condition with a Universal Testing Machine (Zwick Universal testing machine Z005, Ulm, Germany). The size of the specimen was 30 mm × 30 mm × 30 mm (length × width × thickness), and the rate of crosshead movement was fixed at 2 mm/min for each sample. Compressive stress at 20% strain parallel with the foam rise direction was performed according to ASTM D1621-10 [42]. For each compression, five replicate specimens were tested, and the average value was taken along with the standard deviation.

## 4. Conclusions

Microwave-assisted liquefaction was developed for the optimization of the biobased polyols produced from liquefied lignin. Through liquefaction at different microwave heating times and characterization of the resultant polyols, the effect of the microwave heating time on the structure and composition of the polyols was fully investigated. The optimal condition at the microwave heating time (5 min) ensured a high liquefaction yield (97.47%) and polyol with a suitable hydroxyl number (8.628 mmol/g) to be used as a precursor in rigid PU foam synthesis. The liquefied lignin products without pretreatments can be used directly by the one-shot method in the preparation of rigid PU foams. The effect of the [NCO]/[OH] ratio on the mechanical properties of rigid PU foams was also discussed. With the increase of the [NCO]/[OH] ratio from 0.6 to 1.0, their compressive strength was gradually increased. In short, the microwave-assisted liquefaction is a promising heating method to produce lignin-derived polyols. Understanding the liquefied lignin polyol’s yield, composition and molecular structures would be beneficial for the production of the targeted rigid PU foam products.
